# Intraovarian platelet-rich plasma (PRP) infusion appeared to benefit low prognosis IVF patients; however, when compared to controls, no significant benefit could be confirmed

**DOI:** 10.1007/s10815-025-03530-5

**Published:** 2025-06-02

**Authors:** Sarah C. Rubin, Kaleb Noruzi, Emily Chen, Alexis Greene, Martin Keltz

**Affiliations:** 1 New York Medical College, 35 Sunshine Cottage Road, Valhalla, NY 10595 USA; 2 WestMed Reproductive Services, Purchase, NY 10595 USA

**Keywords:** PRP, IVF, PGT-A, Infertility, Euploid

## Abstract

**Purpose:**

To assess whether in-office intraovarian PRP injections among patients with a history of a low euploid embryo yield following freeze-all PGT-A IVF cycle improves transferable embryo yield when compared to controls.

**Materials and methods:**

A retrospective case–control study, between March 2022 and December 2024 where all poor outcome patients who underwent in-office intraovarian PRP injection prior to a repeat freeze-all PGT-A IVF cycle were compared to controls with no intervention between cycles. The primary outcome was transferable embryo yield. Secondary outcomes included: blastocyst yield, aneuploidy rate, and euploid yield.

**Results:**

Thirty-two patients met the inclusion criteria and were compared to 309 controls. Mean age for the PRP and control group were 38.3 ± 3.1 and 38.4 ± 3.7 (*p* = 0.91) respectively. Mean AMH for the PRP group was 1.6 ± 1.1 ng/mL as compared to 1.5 ± 1.0 ng/mL (*p* = 0.34). Following PRP the blastocyst, euploid and transferable embryo yield increased; however, when compared to controls, there were no differences in IVF outcomes. For patients with an AMH ≥ 1, the euploid yield and transferable embryo yield increased by fivefold; however, there was no difference when compared to the control. For patients with an AMH < 1, there were no differences in euploid or transferable embryo yield following PRP.

**Conclusion:**

Although there was an improvement in transferable embryos yielded following PRP, no difference was observed compared to the control group, as the control with a poor initial cycle experienced regression to the mean which may also be a source of bias in the PRP group.

## Introduction

Platelet-rich plasma (PRP) has emerged as a widely explored therapeutic tool in regenerative medicine across multiple medical disciplines, primarily for its regenerative properties that stimulate tissue growth and repair [1, 2]. Its efficacy has been well-documented in areas such as orthopedic surgery and wound recovery [3–6]. PRP first gained attention in reproductive medicine in 2015 for its potential to improve pregnancy outcomes in women with a thin endometrium by promoting endometrial proliferation following intrauterine injection [7]. Despite progress in in vitro fertilization (IVF) and its adjuvants, patients are still susceptible to an age-related decline in fertility and, with that, a lower likelihood of euploid embryos yielded during IVF [2, 8]. To counteract these effects, there has been an investigation into the application of PRP in the ovary [9].

Intraovarian PRP injection was first performed in 2016, largely in patients who met the criteria for primary ovarian insufficiency (POI) and poor ovarian response (POR) [10]. Since then, there have been many studies assessing its efficacy with mixed results. In 2024, Barad et al. conducted a randomized control trial (RCT) where 34 patients with POI received an intraovarian PRP injection into one randomly selected ovary [11]. They found that intraovarian PRP injections into POI ovaries initiated some follicle activation. However, the degree of activation was small; sixty-two percent of treated ovaries produced follicles greater than 4 mm as compared to 26% in untreated ovaries. In 2024, Barrenetxea et al. performed an RCT assessing intraovarian PRP injection in patients with POR [12]. They found that there was no difference in the number of oocytes retrieved, blastocysts, or euploid embryos formed in the control and experimental groups. However, the literature is inconclusive. Éliás et al. in 2024 found in a systematic review that intraovarian PRP improved the number of oocytes retrieved and embryos formed; however, they did not assess for blastulation or euploidy rate [13].

This study aims to evaluate the impact of intraovarian PRP on the yield of transferable embryos, as defined as the sum of euploid and safe to transfer mosaic embryos, in patients undergoing IVF and PGT-A as compared to all equivalent controls. There have been numerous studies on PRP as an intervention for patients with diminished ovarian reserve [14]. However, we studied a population that has a history of less than 2 euploid embryos yielded in prior freeze-all PGTA IVF cycle. We hypothesize that intraovarian PRP may improve the number of euploid and transferable embryos yielded.

## Materials and methods

We conducted a single-institution retrospective case–control study. IRB approval was obtained prior to study initiation. We included all patients who had in-office intraovarian PRP injections between March 2022 and December 2024 following a failed IVF cycle where less than 2 euploid embryos were yielded or with poor ovarian response, where less than 3 oocytes were retrieved. All patients who met these criteria were offered intra-ovarian PRP injection and confirmed that they wanted the procedure. All patients underwent an initial IVF cycle at our institution within 6 months prior to in-office PRP. Each subject underwent at least one subsequent IVF cycle immediately following PRP. All freeze-all PGT-A IVF cycles were within 6 months of the PRP procedure.

Patients were also compared to a control group that included all patients at the same institution between 2018 and 2024 aged 18 to 46 years old who had an AMH less than 4, matched to our study group, and underwent a freeze-all PGT-A IVF cycle. Every patient in the control group underwent an initial and subsequent IVF cycle within 12 months of each other in this time frame and had fewer than 2 euploid embryos yielded in their initial cycle and underwent no new medical or procedural interventions between their first and second PGT-A cycle.

In the menstrual cycle prior to IVF, patients received oral contraceptive pills (OCPs) in preparation for either a Gonadotropin-Releasing Hormone (GnRH) Antagonist or a GnRH Agonist Flare protocol. Protocol and dosing of gonadotropins was based on doctor preference and patient’s prior IVF cycles; total gonadotropin dose is reported in the results. In the post-PRP cycles, the procedure was performed after the completion of menstrual flow and 1–3 days prior to the commencement of controlled ovarian hyperstimulation (COH) for IVF.

All intraovarian PRP injections were performed in-office, in a gynecologic examination room. Patients were premedicated with 10 mg of valium 15–60 min prior to the procedure. Immediately before the procedure, blood samples for autologous PRP were collected by peripheral venipuncture using a 21-G butterfly catheter connected to a vacutainer with negative pressure receiving tubes to obtain a total of 20 mL, between two tubes, of venous blood. Samples were labeled and placed in a centrifuge at room temperature at 3500 RPM for 9 min. Following centrifugation, the blood was fractionated, and the 2 mL of lower-density supernatant was pipetted from the top of the column and placed in a separate clean container. The remaining product in the tube was then discarded. The separated PRP was activated with the addition of 10% calcium carbonate of the volume of supernatant that was collected. One 10 cc syringe was used to draw up the PRP and kept at room temperature. PRP was prepared using a single-spin technique, consistent with those outlined in commercially available kits and prior literature [14, 18, 19].

At the time of the procedure, patients were placed in the dorsal lithotomy position, and the vagina was prepped with sterile povidone-iodine solution. Transvaginal ultrasound was used to assess the position of the ovaries. PRP administration was performed by attaching the syringe to a 17-gauge 35-mm needle inserted through a transvaginal ultrasound needle guide. The ovaries were visualized using ultrasound guidance and the ovaries were aligned with the needle guide to prevent harm to nearby structures. The needle was then advanced with puncture of the central ovarian stroma and 2–4 mL of PRP sample was injected in multiple stromal locations with visualization of the iliac vein and artery throughout the procedure. This procedure was repeated for the contralateral ovary. After PRP injection was completed, transvaginal ultrasound was repeated to ensure that there was no disruption of nearby vascular structures and no free pelvic fluid present. The procedure in total was completed in 10 min or less. No local anesthetic was used during the procedure. Patients were observed for 10–15 min following the procedure then discharged home.

For both the pre- and post-PRP cycles, COH was initiated with exogenous gonadotropins. The dose of gonadotropins, recombinant FSH (rFSH) and/or human menopausal gonadotropins (HMG), was individually adjusted based on ovarian response. Transvaginal sonography and serial estradiol (E2) levels were used to monitor ovarian follicular development. Once a dominant follicle reached a diameter of 18 to 20 mm, 2500–10,000 IU of hCG was administered based upon the patient’s individual weight and risk for OHSS. Transvaginal ultrasound-guided oocyte retrieval was performed 36 h later. Insemination was performed with either standard insemination or intracytoplasmic sperm injection (ICSI) based on male factor indications. Fertilization was confirmed 20 h later by the presence of two pronuclei (2PN). Embryos were kept in culture until day 3–4, when they underwent laser-assisted hatching. Day 5–7 blastocysts underwent trophectoderm biopsy and were subsequently frozen. Biopsy samples were sent to CooperGenomics (CooperSurgical Fertility Solutions, Livingston, NJ) for PGT-A testing, which reported back NGS data.

The first IVF cycle following PRP was compared to the IVF cycle immediately prior to PRP. The primary outcome was the number of transferable embryos per cycle, defined as the sum of euploid and “transferable” mosaic embryos. Of note, our prior studies have shown the transfer of mosaic embryos has non-inferior live birth outcomes when compared to euploid embryos [15]. Secondary outcomes included: the number and percentage of blastocysts, aneuploidy rate, and euploid yield. Patients in the study and control group were compared regardless of AMH, then compared to the control based on an AMH of greater than or equal to 1 ng/mL and an AMH of less than 1 ng/mL. This stratification was conducted to assess whether PRP benefits patients who may meet criteria for diminished ovarian reserve. Statistical significance in our primary outcome used *p* ≤ 0.05, while our secondary outcomes required *P* ≤ 0.01 due to multiple significance testing. Parametric continuous data among the same patients in their first and second cycles were compared using the paired *t*-test. Where there was no normal distribution, a non-parametric Wilcoxon signed rank test was employed. To compare the IVF outcomes for the PRP versus the control group, a delta was calculated as the difference between the second and first IVF cycle for each group and then compared with Wilcoxon rank sum test. A power analysis to rule out false negatives in this study which demonstrated no difference between the delta of transferable embryos in the PRP group and controls was performed; the study was powered to detect a difference of 0.76 transferable embryos between the delta of the PRP and control groups at a power of 80% and significance level of 0.05. All data were analyzed using R programming software.

## Results

Thirty-two patients met the inclusion criteria for this study, while 309 controls were included. The PRP group had a mean age at the time of first visit of 38.3 $\pm$ 3.1 years as compared to the control group with a mean age of 38.4 ± 3.7 (*p* = 0.91) as shown in Table 1. There was no difference in mean Anti-Müllerian Hormone (AMH) level between the PRP group and the control group with 1.6 ± 1.1 ng/mL as compared to 1.5 ± 1.0 ng/mL (*p* = 0.34). Patients in the PRP group and control group had no differences in maximum FSH with 7.4 ± 2.8 mIU/mL and 7.9 ± 4.1 mIU/mL, respectively (*p* = 0.4). In the first IVF cycle, patients had a total gonadotropin dose in the PRP group and control of 4881 ± 1570 IU and 5423 ± 1443 IU, respectively (*p* = 0.05). In the second cycle, the PRP group and controls had a total gonadotropin dose of 4798 ± 1457 IU and 5388 ± 1530 IU, respectively (*p* = 0.04). Additionally, there were no differences in dosing between the first and second cycle for the PRP group and control groups individually. For the control group, 81.6% (252/309) had ICSI performed for their first IVF cycle as compared to 84.4% (27/32) in the PRP group (*p* = 0.81). In the second cycle, 83.5% (258/309) of patients in the control used ICSI as compared to 93.8% (30/32) in the PRP group (*p* = 0.2).

**Table 1:** Patient baseline characteristics

Parameter	PRP group Mean (SD)	Control group Mean (SD)	*p*-value
Age	38.3 (3.1)	38.4 (3.7)	0.91
AMH (ng/mL)	1.6 (1.1)	1.5 (1.0)	0.34
FSH max (mIU/mL)	7.4 (2.8)	7.9 (4.1)	0.4
Total gonadotropin dose (IU) First cycle Second cycle	4881 (1570) 4798 (1457)	5423 (1443) 5388 (1530)	0.05 0.04
ICSI performed (%) First cycle Second cycle	84.4% (27/32) 93.8% (30/32)	81.6% (252/309) 83.5% (258/309)	0.81 0.2
Male factor infertility (%)	53% (17/32)	32% (100/309)	0.03*
Diminished ovarian reserve (%)	34% (11/32)	38% (118/309)	0.8
History of endometriosis (%)	16% (5/32)	8% (25/309)	0.18
Tubal disease (%)	6.3% (2/32)	10% (31/309)	0.75
Uterine factor (%)	3.1% (1/32)	3.6% (11/309)	1
Recurrent pregnancy loss (%)	3.1% (1/32)	5.8% (18/309)	1
PCOS (%)	0% (0/32)	6.5% (2/309)	1
Unexplained infertility (%)	15.6% (5/32)	14.6% (45/309)	0.8

* statistically significant

For male factor infertility in the control and PRP group 32% (100/309) and 53% (17/32) patients had this diagnosis respectively (*p* = 0.03) as shown in Table 1. There were no differences in the number of patients with Diminished Ovarian Reserve with 38% (118/309) in the control group and 34% (11/32) in the PRP group (*p* = 0.8). There were no differences in the number of patients with a history of endometriosis in the PRP group as compared to the control (16% (5/32) vs 8.0% (25/309), *p* = 0.18). There were no differences in the number of patients with Tubal Disease for the PRP and control group with 6.3% (2/32) and 10% (31/309), respectively (*p* = 0.75). In the control group 3.6% (11/309) had uterine factor, while in the PRP group 3.1% (1/32) had uterine factor (*p* = 1). In the PRP group, 3.1% (1/32) had recurrent pregnancy loss, while 5.8% (18/309) for the control group (*p* = 1). No patients in the PRP group had a diagnosis of PCOS, but 6.5% (2/309) in the control group had PCOS (*p* = 1). There were no differences in the number of patients who had unexplained infertility with 15.6% (5/32) in the PRP group and 14.6% (45/309) in the control group (*p* = 0.8).

When analyzing all patients who underwent intra-ovarian PRP, there was no difference in the number of oocytes retrieved prior to and following PRP at 7.0 ± 4.1 and 7.4 ± 5.7 (*p* = 0.55). There was no statistical difference in the number of 2PN embryos following PRP from 4.3 ± 3.5 to 5.2 ± 4.9 (*p* = 0.188) as shown in Table 2. Also, the number of blastocysts increased following PRP. Intraovarian PRP was also associated with an increase in the yield of euploid embryos from 0.19 ± 0.4 to 0.75 ± 1.1 (*p* = 0.01). There was also an increase in the number of transferable embryos from 0.21 ± 0.42 to 0.91 ± 1.3 (*p* = 0.01). There were no significant improvements in euploidy rate or transferable embryo rate. There were also no statistical differences in the aneuploidy rate or aneuploid embryo yield, although the aneuploidy rate trend decreased, however was not statistically significant.

**Table 2:** Paired outcomes for IVF cycles immediately before and after intraovarian PRP injection as compared to the control

	PRP group *n* = 32			Control group *n* = 309		
Outcome measure	Pre-PRP mean (SD)	Post-PRP mean (SD)	Confidence interval, *p*-value	First cycle mean (SD)	Second cycle mean (SD)	Confidence interval, *p*-value
2PN yield	4.3 (3.5)	5.2 (4.9)	[− 2.3, 0.47] 0.188	4.5 (3.6)	5.5 (4.2)	[− 1.4, − 0.5] < 0.001*
Blastocysts yield	1.6 (1.7)	2.5 (2.7)	[− 1.7, − 0.22] 0.01*	1.6 (1.6)	2.3 (2.3)	[− 1.0, − 0.5] < 0.001*
Percent blastocysts	37% (34)	49% (34)	[− 0.28, 0.05] 0.15	36% (32)	39% (31)	[− 0.08, 0.01] 0.14
Euploid yield	0.19 (0.4)	0.75 (1.1)	[− 1.0, − 0.13] 0.01*	0.26 (0.44)	0.8 (1.2)	[− 0.7, − 0.4] < 0.001*
Percent euploid	11% (27)	23% (31)	[− 0.27, 0.05] 0.16	14% (28)	24% (34)	[− 0.15, − 0.06] < 0.001*
Transferable embryo yield	0.21 (0.42)	0.91 (1.3)	[− 1.2, − 0.17] 0.01*	0.5 (0.7)	1.0(1.5)	[− 0.7, − 0.4] < 0.001*
Percent transferable	13% (28)	27% (36)	[− 0.32,0.04] 0.11	23% (35)	32%(39)	[− 0.14, − 0.04] < 0.001*
Aneuploid yield	1.3 (1.75)	1.4 (1.9)	[− 0.6,0.4] 0.7	1.1 (1.2)	1.2(1.5)	[− 0.33, 0.02] 0.1
Percent aneuploid	54% (48)	42% (43)	[− 0.1, 0.3] 0.3	49% (44)	43% (42)	[− 0.009, 0.11] 0.1

* statistically significant

When analyzing IVF outcomes for our control group by comparing their first and second IVF cycles, there was a statistical improvement in the number of oocytes retrieved, 8.1 ± 5.9 to 8.9 ± 6.6 (*p* = 0.005), and an increase in the number of 2PN per cycle from 4.5 ± 3.6 to 5.5 ± 4.2 (*p* < 0.001) in the subsequent cycle as shown in Table 2. In our control, there was also improvement in the number and percentage of euploid blastocysts. There was an improvement in the yield and percentage of transferable embryos. There were no differences in aneuploidy yield or rate.

When comparing our control group to our PRP group, there were no statistically significant differences in IVF outcomes as shown in Table 3. When comparing euploid yield and percentage, the addition of PRP showed no improvement in outcomes. This held true when comparing the percent and yield of transferable embryos, aneuploid embryos and blastocysts.

**Table 3:** Delta comparison: change from first to second IVF cycle, PRP group compared to controls

Outcome measure	Second cycle change in PRP Mean (SD)	Second cycle change in control Mean (SD)	*p*-value
2PN yield	+ 0.9 (3.8)	+ 0.9 (3.8)	0.97
Blastocyst yield	+ 1.0 (2.1)	+ 0.7 (2.2)	0.5
Blastulation rate	+ 12% (46)	+ 3.4% (41)	0.27
Euploid yield	+ 0.6 (1.2)	+ 0.5 (1.2)	0.9
Euploidy rate	+ 11% (44)	+ 10.7% (41)	0.96
Transferable embryo yield	+ 0.7 (1.4)	+ 0.6 (1.5)	0.67
Transferable rate	+ 14% (50)	+ 9.0% (45)	0.55
Aneuploid yield	+ 0.09 (1.4)	+ 0.15 (1.6)	0.91
Aneuploidy rate	− 13% (58)	− 5.2% (55)	0.47

IVF outcomes were evaluated as stratified by AMH levels. For patients who received intraovarian PRP, 11 had an AMH less than 1 ng/mL while 21 had an AMH greater than or equal to 1. For our control group, there were a total of 184 patients with an AMH greater than or equal to 1 and 125 patients with an AMH less than 1.

For those with an AMH greater than or equal to 1, the average age of patients in our PRP group was 37.9 ± 3.9 as compared to 37.8 ± 3.3 in our control group (*p* = 0.6). For our PRP group, there was an increase in blastocyst yield as shown in Table 4. There was also an increase in the euploid and transferable embryo yield. There was no decrease in the aneuploid yield or aneuploidy rate in our PRP group. In the comparison between our control and PRP group with an AMH greater than or equal to 1 there were no significant differences in any IVF outcomes as shown in Table 5.

**Table 4:** Paired outcomes for IVF cycles immediately before and after intraovarian PRP injection for AMH < 1 and AMH $\ge$ 1

	AMH < 1 *n* = 11			AMH$\ge$1 *n* = 21		
Outcome measure	Pre-PRP mean (SD)	Post-PRP mean (SD)	Confidence interval, *p*-value	Pre-PRP mean (SD)	Post-PRP mean (SD)	Confidence interval, *p*-value
2PN yield	2.7 (1.7)	2.7 (2.8)	[− 1.5, 2.0] 0.89	5.0 (4.0)	6.4 (5.3)	[− 4.0, 1.0] 0.15
Blastocyst yield	1.2 (0.9)	1.4 (1.4)	[− 1, 1] 0.8	1.8 (2.0)	3.1 (3.1)	[− 3.5, − 0.5] 0.01*
Blastulation rate	44% (34)	48% (36)	[− 0.4, 0.3] 0.82	34.1% (34)	50% (34)	[− 0.4, 0.03] 0.09
Euploid yield	0.2 (0.4)	0.1 (0.3)	[− 0.1, 0.3] 0.34	0.19 (0.4)	1.1 (1.2)	[− 1.5, − 0.3] 0.005*
Euploidy rate	12% (31)	3.0% (10)	[− 0.1, 0.3] 0.34	11% (26)	32.7% (34)	[− 0.6, − 0.02] 0.03*
Transferable embryo yield	0.2 (0.4)	0.2 (0.4)	[− 0.3, 0.3] 1	0.24 (0.44)	1.3 (1.5)	[− 1.8, − 0.3] 0.008*
Transferable rate	12% (31)	6.1% (13)	[− 0.2, 0.3] 0.55	13.5% (27)	38% (39)	[− 0.7, 0.0] 0.05*
Aneuploid yield	1.0 (0.8)	1.0 (1.3)	[− 2.0, 1.5] 0.9	1.4 (2.1)	1.6 (2.2)	[− 1.0, 1.0] 0.6
Aneuploidy rate	70% (46)	48% (48)	[0.0,1] 0.32	46% (47)	38% (40)	[− 0.3, 0.6] 0.6

* statistically significant

**Table 5:** Delta comparison: change from first to second IVF cycle, PRP group compared to controls. Patients with an AMH < 1 and AMH greater than or equal to 1

	AMH < 1			AMH $\ge$ 1		
Outcome measure	Second cycle change in PRP Mean (SD)	Second cycle change in control Mean (SD)	*p*-value	Second cycle change in PRP Mean (SD)	Second cycle change in control Mean (SD)	*p*-value
2PN yield	+ 0 (2.7)	+ 0.6 (2.8)	0.4	+ 1.4 (4.3)	+ 1.1 (4.4)	0.79
Blastocyst yield	+ 0.2 (1.4)	+ 0.48 (1.8)	0.5	+ 1.4 (2.2)	+ 0.85 (2.4)	0.27
Blastulation rate	+ 4% (56)	+ 1% (47)	0.88	+ 16% (40)	+ 5.1% (37)	0.24
Euploid yield	− 0.09 (0.3)	+ 0.2 (0.77)	0.23	+ 0.9 (1.3)	+ 0.76 (1.4)	0.4
Euploidy rate	− 9% (30)	+ 5% (41)	0.38	+ 22% (47)	+ 15% (40)	0.56
Transferable embryo yield	+ 0 (0.44)	+ 0.2 (1.0)	0.6	+ 1.04 (1.6)	+ 0.82 (1.6)	0.49
Transferable rate	− 6% (33)	+ 3.2% (45)	0.7	+ 25% (55)	+ 13% (46)	0.41
Aneuploid yield	+ 0 (1.3)	+ 0.3 (1.4)	0.4	+ 0.14 (1.4)	+ 0.05 (1.7)	0.62
Aneuploidy rate	− 21% (60)	+ 2.5% (60)	0.2	− 8% (57)	− 11% (52)	0.62

For those with an AMH less than 1, the average age of patients in our PRP group was 39.1 ± 3.5 as compared to 39.3 ± 2.6 (*p* = 0.97). When assessing all IVF outcomes, there was no improvement in euploid yield for the PRP group with a prior and post-PRP yield of 0.2 ± 0.4 and 0.1 ± 0.3 (*p* = 0.34) as shown in Table 4. There was also no change in transferable embryo yield following PRP, 0.2 ± 0.4 and 0.2 ± 0.4 (*p* = 1). In the comparison of our control and PRP group with an AMH less than 1, there were no significant differences in any IVF outcomes as shown in Table 5.

## Discussion

In this case–control study, in-office intraovarian PRP injection was associated with an improved euploid and transferable embryo yield. However, when comparing the PRP group to controls, there were no significant differences in euploid, transferable embryo, or blastocyst yield, so the benefits were no longer seen as both the treatment and control had improvements due to regression to the mean in their outcomes. For patients with an AMH greater than or equal to 1, the euploid yield and transferable embryo yield increased; however, there was no significant difference when compared to the control. For patients with an AMH < 1, there was no difference in yield following PRP due to regression to the mean. Any perceived improvement in outcomes following PRP was a result of selecting patients with a poor response in their first cycle, for both the PRP and control group, who had returned to a median performance in their second cycles.

Multiple cohort studies have shown an increase in oocyte count following PRP in analysis of pre-post cycles [20–23]. However, none of these studies are well controlled. They further encourage the use of PRP without accounting for regression to the mean, possibly introducing unnecessary interventions and undue risk to the patient.

Alternatively, there are studies on intraovarian PRP with similar findings to our current study that are well controlled. In 2024, Herlihy et al. conducted a multi-center randomized control trial assessing intraovarian PRP in DOR patients [16]. There were no differences in mature oocytes retrieved per cycle or blastocysts between the PRP and control groups. Similarly, the study found no improvement in euploid blastocyst yield; there were 0.8 ± 1.1 euploid embryos yielded in the cycle following PRP similar to our PRP group with 0.8 ± 1.1 euploid embryos in their second cycle.

In 2025, Yu et al. conducted a case–control study assessing intraovarian PRP and IVF outcomes and found that there was significant improvement in good quality blastocysts following intraovarian PRP; they did not assess PGT-A tested embryos; however, this is inconsistent with our study’s findings [17]. They additionally found that the most notable benefits with PRP was within 1 to 2 months post-injection, which is when our post-PRP cycles were performed. However, in their study, they prepared PRP using 60 mL of whole blood which is a higher volume than the 20 mL of whole blood collected in our study. Therefore, further investigation into the time-related benefits and optimal volume needed for PRP should be considered in assessing its efficacy. It is possible that our study had negative findings due to the lower volume of PRP administered.

Our study has several limitations, with the most notable being the small sample size. In addition, our study had a low power, which may limit the generalizability of our findings. A larger multi-center trial is needed to assess whether these results apply to a broader population of patients with a poor prognosis undergoing IVF. A further limitation is the retrospective nature of the study which can increase the risk of bias in who was offered intraovarian PRP as compared to patients in the control group. An additional limitation of this study is seen in the differences in baseline characteristics; more patients who received intraovarian PRP had male factor infertility when compared to the control group which may limit the interpretation of these results. However, the majority of patients in this study had ICSI performed, thereby decreasing the effect on male factor infertility on patient outcomes. Furthermore, when comparing the number of oocytes retrieved between the PRP and control groups, there were no differences. The lack of differences in oocyte count in addition to poor embryo development, is reflective of poor oocyte response to PRP and not due to male factor infertility alone. The only explanation for the post-PRP IVF outcomes in our patient population is regression to the mean. Despite these limitations, our study has several strengths. Conducting the research at a single institution allowed for consistency in the administration of the intraovarian PRP procedure and facilitated close monitoring of patient progress and IVF outcomes. However, to further validate these findings and assess the long-term clinical benefits of incorporating intraovarian PRP into IVF treatment protocols, larger, prospective studies with randomized designs are needed.

In our retrospective case–control study, we demonstrated that intraovarian PRP injection did not improve IVF outcomes when compared to controls. However, future prospective studies with randomization and larger sample sizes are warranted to assess any benefit of in-office intraovarian PRP in our low prognosis IVF population.

## Conclusion

Although there was an improvement in blastocyst formation and euploid yield following PRP, no significant difference was observed compared to the control group. Thus, any observed improvement is likely due to regression to the mean rather than a direct benefit of PRP.

## Data Availability

The original contributions presented in this study are included in the article. Further inquiries can be directed to the corresponding author.
